# Collagen Structure, Synthesis, and Its Applications: A Systematic Review

**DOI:** 10.7759/cureus.24856

**Published:** 2022-05-09

**Authors:** Mahesh Shenoy, Nishath Sayed Abdul, Zeeshan Qamar, Bader Musfer Al Bahri, Khalid Zuhair K Al Ghalayini, Ateet Kakti

**Affiliations:** 1 Department of Oral Pathology, College of Dentistry, Riyadh Elm University, Riyadh, SAU; 2 Department of Oral Biology, College of Dentistry, Riyadh Elm University, Riyadh, SAU; 3 Department of Restorative Dentistry, Consultant Ministry of Health, Riyadh, SAU; 4 Department of Endodontics, Consultant Ministry of Health, Riyadh, SAU; 5 Department of Pediatric Dentistry, College of Dentistry, Riyadh Elm University, Riyadh, SAU

**Keywords:** systematic review, applications, synthesis, structure, collagen

## Abstract

Resorbable collagen has been utilized to treat wounds, close graft, and tooth extraction sites, and enhance recovery. Collagen-based membranes are also used as barriers in periodontal and implant therapy to limit epithelial migration and allow cells with the regenerative capacity to fill the problem area. This systematic review was carried out to analyze the studies focusing on collagen structure, synthesis, and its applications. A detailed and extensive search was performed with the help of the keywords "collagen structure", "collagen synthesis" and "collagen applications". There was extensive literature search in reliable and authentic databases like PubMed, Scopus, Web of Sciences, Ovidsp, and Cochrane library to obtain papers focusing on collagen structure, synthesis, and applications. During the systematic review, data were obtained concerning the following parameters. Type of study, nature of aim of the study, size of the sample in the study, gender and age of the subjects included in the study, prevalence of skin diseases where collagen was used for treatment, dose of collagen used, form in which collagen was used, the origin of collagen used, analysis of different variables, structure, and synthesis of collagen. Twenty-two studies were included in this systematic review. The studies discussed the structure, synthesis, and applications of collagen in treatment. In studies focusing on the application of collagen supplements, most of the study subjects were females (68.3%). The study subjects included both healthy and unhealthy subjects. The study subjects were divided into two categories. One category was the intervention group, while another group was the placebo group. Collagen was administered in hydrolysate form (90%) in some studies, bovine form (2.3%), and porcine form (3.4%) in other studies. Collagen supplementation was found to provide better results in both healthy and unhealthy effects in improving the health of skin, cornea, bone, periodontium, face, etc. It can be concluded that collagen is an integral part of the body. The application of collagen supplements can be pretty effective in maintaining the proper health of several important structures of the body like skin, face, cornea, nails, periodontium, etc. Thus, a detailed study of the molecular structure of collagen and genes associated with each type of collagen is essential for further research and treatment of collagen-associated disorders.

## Introduction and background

Collagen is a principal protein of connective tissue. When collagen was first characterized as "that component of connective tissue, which gives gelatin on boiling," the Greek word "kolla" (glue) and a French word "collagen" were used to describe the glue-producing ingredient of connective tissue. Collagen is also the most abundant protein in mammals, a major component of connective tissue, accounting for around 25% of total protein content. Because of its great tensile strength, this material is often used to construct ligaments and tendons. Collagen is an extracellular matrix component in all dental tissues save the enamel. Collagen is found in bones, cartilage, and teeth. Collagen also fills out the cornea, which is present in the crystalline form [1-3].

As of this writing, there are at least 29 distinct kinds of collagen known to science. They are grouped into three categories based on their ability to generate fibrils. They are referred to as "fibril-forming colloids" because they produce banded fibrils and are found in the collagen types I through VIII [4-6]. This group of collagens contains kinds IX, XII, XIV, and potentially even IX, XVI, XV, XVI, XVIII, & XVII, and types XVI, XVII, XVII, XXVI, & XXVII as well. Types IV, VIII, and X of network-forming collagens, types VI and VII of beaded collagens, types VI and VII of anchoring fibrils, and invertebrate cuticle collagens, comprise the third category of non-fibrillar collagens. They produce sheets of protein membranes around tissues and organisms. Deterioration of this protein causes wrinkles as we get older because of its role in the skin's strength and flexibility [7-10].

Dental, orthopedics and surgical procedures utilize collagens to fabricate artificial skin replacements to treat severe burns. Pharmaceutical, aesthetic, and prolotherapy use collagen (strengthening the lax ligaments). Blood coagulating cotton textiles, injections to treat soft tissue abscesses; dental bone filling materials; and a permeable membrane for periodontal regeneration are examples of how collagen may be used in therapy. When collagen is manufactured, it may take the form of cross-linked solids or gels with a lattice-like structure [11-14]. The use of resorbable collagen in dressings, graft closure, and tooth extraction sites, among other applications, dates back to the 1970s. As a barrier preventing epithelial migration and allowing cells with regeneration ability into the defect region, collagen-based membranes have been employed in periodontal and implant treatment [15,16]. This systematic review was carried out to analyze the studies focusing on collagen structure, synthesis, and its applications.

## Review

Design and methods

*Inclusion Criteria* 

Those published papers were selected that fulfilled the following criteria: 1) Papers that reflected structure, synthesis, and applications of collagen for treatment purposes. 2) Papers that included the subjects in collagen were used alone for treatment in their study. 3) Papers that were published in the English language only.

Exclusion Criteria

Those papers were not selected that had the following features: 1) Papers focused application of collagen and other supplements in the management of diseases. 2) The literature was published in non-commercial formats, like the abstract of the conference.

Literature Search

**Figure 1:**
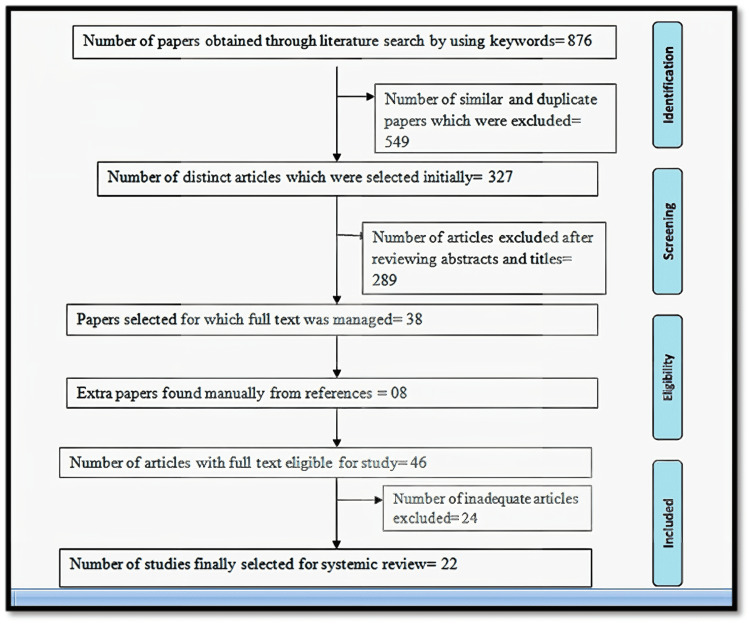
Representation of selection of articles for systematic review

A detailed and extensive search was performed with the help of the keywords "collagen structure", "collagen synthesis", and "collagen applications". There was extensive literature search in reliable and authentic databases like PubMed, Scopus, Web of Sciences, Ovidsp, and Cochrane library for obtaining papers focusing on the structure, synthesis, and applications of collagen [17]. A total of 876 papers were found. After that, 549 papers were removed that were similar or duplicate articles. Initially, there was a selection of 327 different papers. Then after there was reviewing of abstracts and titles of papers. Two hundred and eighty-nine papers were excluded after this review. Finally, 38 papers were selected that wholly fulfilled the inclusion and exclusion criteria. Then complete text of these 108 papers was managed. Eight more articles with full text were obtained from the references of the article. The final review was carried out, and 24 more papers were eliminated. Hence finally, 22 articles with full text were included in this systemic review. (Figure 1)

Data Extracted

During the systematic review, data were obtained concerning the following parameters. Type of study, nature of aim of the study, size of the sample in the study, gender and age of the subjects included in the study, prevalence of skin diseases where collagen was used for treatment, dose of collagen used, form in which collagen was used, the origin of collagen used, analysis of different variables.

Statistical analysis

SPSS Inc. SPSS for Windows, Version 14.0. Chicago, SPSS Inc. software was used for carrying out a systematic review analysis. A comparison of variables among the groups was carried out with the help of independent sample t-tests. In contrast, a comparison of treatment effects among groups was carried out with the help of chi-square tests. The difference among the means of groups was represented with t-test confidence intervals, while chi-squared confidence intervals represented differences among the population.

Results

Most of the publications (98.7%) were published after 2018. The papers reflected data from 25 countries. The studies included in this systematic review were from worldwide populations present in Asia, Africa, Europe, and the USA. Most of the studies included focusing on applications of collagen were randomized controlled trials (90%). Among them, 23% of papers were prospective, while 69% were retrospective in nature. It was found that a maximum number of articles had descriptive aims and objectives.

The studies discussed the structure, synthesis, and applications of collagen in treatment. In studies focusing on the application of collagen supplements, most of the study subjects were females (68.3%). The study subjects included both healthy and unhealthy subjects. The study subjects were divided into two categories. One category was the intervention group, while another group was the placebo group. Collagen was administered in hydrolysate form (90%) in some studies, bovine form (2.3%), and porcine form (3.4%) in other studies. When there was an analysis of different variables like facial moisture, skin elasticity, facial elasticity, nail ceramides, and nail sphingosine, there was an improvement in the intervention group compared with the placebo group. There was a decrease in some parameters in intervention groups compared to placebo groups, like the hardness of skin, periorbital wrinkles, dryness of skin, facial dryness, hardness of nails, etc.

According to most research, there are three peptide chains in the collagen structure: 1. Rigid protein with 300 Kilo Dalton (kDa) molecular weight, length of 300 nm, and width of 1.5 nm is found in vertebrates 3,000 amino acids make up the molecule in its entirety "Madras Triple Helix Geometry" refers to the collagen triple helix coil structure. 2. 300 nm right-handed coil, helix radius 2.8 nm, the molecular diameter of 1.5 nanometers, and coil pitch 85.5 nanometers. 3. 200 nm right-handed coil, helix radius 1.8 nm. Under a light microscope, the collagen fibres are found to be structured in various ways in different tissues, such as in tendons where they are placed in parallel bundles and in the skin, where the bundles run in varied directions but are mainly parallel to the surface.

Collagen supplementation was found to provide better results in both healthy and unhealthy effects in improvement of the health of skin, cornea, bone, periodontium, face, etc.

Discussion

Uses for collagen include blood coagulating cotton textiles, injectable therapies for soft tissue abscesses, dental bone filling materials, and a porous membrane for periodontal regeneration. A lattice-like structure may be achieved by cross-linking collagen to form solids or gels. With the use of resorbable collagen has been utilized to repair wounds such as closure grafts and extraction sites and improve recovery. Additionally, collagen membranes have been employed to treat periodontal disease and dental implants as a barrier to epithelial migration. This systematic review was carried out to analyze the studies focusing on collagen structure, synthesis, and applications.

Numerous studies reveal that collagen has a peptide chain structure composed of three interlocking strands. It has a molecular weight of 300 kilodaltons and a length, breadth, and thickness of 300 nm (kDa). The total number of amino acids in the molecule is around 3,000. Madras Triple Helix Geometry are three distinct aspects of the structure of collagen 300 nm-long right-handed coils, a 1.5-nm molecular diameter of the triple helix, has an overall pitch of 85.5 coils per inch, and the 85.5 coil pitch.

The arrangement of collagen fibres varies depending on the tissue from which it is derived. Tendon fibres are arranged in parallel bundles, whereas skin fibres are scattered throughout the surface [18-20].

Connective tissue is made up mostly of collagen. Collagen is derived from the Greek word "kolla" (glue) and the French term "collagen" and was initially described as "that constituent of connective tissue that provides gelatin when cooked." About a quarter of the total protein in mammals is collagen, which is the most abundant protein in the body and a key component of connective tissue. Because of its great tensile strength is a crucial component in ligaments and tendons throughout the human body. Dentin, pulp, and other tooth tissues save enamel, including collagen in their extracellular matrix. Bone, cartilage, and teeth are all made of collagen [21-24].

There are at least 29 different forms of collagen in the collagen family. They are categorized into three groups based on their capacity to produce fibrils. Banded fibrils are formed by fibril-forming collagens, which are collagen types I, II, III, V, XI, XXIV, and XXVII. Non-collagenous sequences are found attached to the surface of fibril-producing collagens in the second category of collagens, which includes collagens with collagenous domains interrupted by non-collagenous sequences such as types IX, XII, XIV, and maybe XVI, XIX, XX, XXI, XXII, XXIII, and XXVI [25-28]. The details of the included articles are shown in a table (Table 1).

**Table 1:** Important details of the studies included in this systematic review DCSS: diffuse cutaneous systemic sclerosis. MRSS: modified Rodnan skin thickness score. CP: collagen peptide. TEWL: trans-epidermal water loss. BCP: bioactive collagen peptide. LMWCP: low molecular weight collagen peptide. H-CP: higher collagen peptide. L-CP: lower collagen peptide

Details of Authors	Details about Subjects (n)	Details of Groups of study subjects	Details about origin of collagen, form of collagen and dose of collagen	Duration of study	Analysis of Results	Variables analysed
Postlethwaite and associates in year 2008 in population of USA [13]	DCSS patients (n = 168)	Intervention: type I collagen (n = 83) Placebo: acetic acid (n = 83)	Bovine/intact/500 µg per day	12 months	Decrease in late-phase DCSS compared with placebo	MRSS
Choi and associates in year; 2014 in population of the South Korea [25]	Healthy subjects	Group A: no supplement (n = 8) Group B: CP (n = 8) Group C: CP + vitamin C (n = 8) Group D: vitamin C (n = 8)	Hydrolysate/CP = 3 g and vitamin C = 500 µg	12 weeks	Increase in CP group compared with controls	Stratum corneum hydration
					Decrease in CP group compared with controls	TEWL
					Increase in CP group compared with controls	Skin elasticity
Kuwaba and associates in year 2014 [26]	Women with dry and saggy face	Intervention: CP/Placebo:	Fish/hydrolysate/5 g	8 weeks	Decreased compared with placebo group	Wrinkle number
					Increased compared with placebo group	Skin dryness
Proksch and associates in year 2014 in population of Brazil [2]	Healthy females (n = 57)	Intervention: BCP Placebo: maltodextrin	NR/hydrolysate/2.5 g per day	8 weeks	Decreased compared with placebo group	Skin wrinkle volume
					Increased compared with placebo group	BCP type I procollagen
					Increased compared with placebo group	BCP elastin
Inoue and associates in year 2015 in population of China [14]	Healthy females	Intervention 1: H-CP (n = 28) Intervention 2: L-CP (n = 29) Placebo: maltodextrin (n = 28)	Fish gelatin/hydrolysate/5 g	8 weeks	Increase in H-CP group compared with L-CP and placebo; increase in L-CP group compared with placebo	Facial moisture
					Increase in H-CP group compared with L-CP and placebo.	Facial elasticity
					Decrease in H-CP group compared with L-CP and placebo; decrease in L-CP group compared with placebo	Facial roughness
Sugihara and associates in 2015 in population of China [27]	Healthy females	Intervention: CP (n = 28) Placebo: maltodextrin (n = 28)	hydrolysate/2.5 g	8 weeks	Increased compared with placebo group	Facial hydration
					Increased compared with placebo group	Facial elasticity
					Decreased compared with placebo group	Facial roughness
Mori and associates in year 2017in population of Japan [12]	Healthy females with nail fragile and or thinly peeled off	Intervention: CP (n = 10) Placebo: dextrin (n = 10)	Porcine skin/hydrolysate/5 g	12 weeks	Increased compared with placebo group	Nail moisture
					Decreased compared with placebo group	Nail hardness
					Increased compared with placebo group	Nail sphingosine
					Increased compared with placebo group	Nail ceramides
Kim and associates in year 2018 in population of Korea [3]	Healthy females	Intervention: LMWCH (n = 32) Placebo: same formula except CP	Fish/hydrolysate/1 g	12 weeks	Increase in LMWCH group compared with Placebo	Skin hydration
					Decrease in LMWCH group compared with placebo	Crow's-feet scores
					Increase in LMWCH group compared with placebo	Skin elasticity
Koizumi and associates in year 2018 in population of Japan [15]	Healthy females	Intervention: beverage containing CP (n = 38) Placebo: beverage	Fish/hydrolysate/3 g	12 weeks	Increased compared with placebo group	Facial moisture
					Increased compared with placebo group	Skin elasticity
					Decreased compared with placebo group	Periorbital wrinkles
Yamamoto [28] and associates in year 2018 and population of Japan	Healthy subjects with dry skin	Intervention: drink containing CP (n = 18) Placebo: drink (n = 18)	Porcine skin/hydrolysate/10 g	8 weeks	Decreased compared with placebo group	TEWL

Notably, non-fibrillar collagens include networked, beaded, and anchoring fibrils and invertebrate cuticle collagens. Sheets or protein membranes surrounding tissues and organisms are made up of collagens. Skin elasticity and firmness are dependent on this protein, which degenerates with age. It is common to practice to employ collagen in the production of artificial skin replacements for burn victims and a range of dental, orthopedic, and surgical applications. Collagen is a protein that is used in a wide range of goods, including those for medical use, cosmetics, and prolotherapy [29-31].

The majority of the publications (98.7%) included in this systematic review were published after 2008. Data from 25 countries was reflected in the publications. The studies in this systematic review came from communities worldwide, including Asia, Africa, Europe, and the United States. The majority of the studies focusing on collagen applications were randomized controlled trials (90%). Twenty-three percent of the articles were prospective, while 69% of the papers were retrospective. According to the findings, the majority of articles had descriptive purposes and objectives.

Collagen's structure, production, and therapeutic applications were discussed in the studies. Most study volunteers in collagen applications studies were females (68.3%), and they were divided into two groups [32-34]. The intervention group was one category, while the placebo group was another. In specific experiments, collagen was given in hydrolysate form (90%), bovine form (2.3%), and porcine form (3.4%). The intervention group outperformed the placebo group when different factors were analyzed, such as facial moisture, skin flexibility, facial elasticity, nail ceramides, and nail sphingosine [35,36].

Some criteria, such as skin hardness, periorbital wrinkles, dryness of skin, face dryness, and nail hardness, decreased in intervention groups compared to placebo groups. Collagen supplementation was found to improve skin ageing parameters in both good and bad ways.

## Conclusions

It can be concluded that collagen is an integral part of the body, and the application of collagen supplements can be quite effective in maintaining proper health of several important structures of the body like skin, face, cornea, nails, periodontium, etc. Collagen is the main protein of connective tissue in animals and the most abundant protein in mammals, making up about 25% of the total protein content. It has great tensile strength and is the main component of ligaments and tendons. Collagen is capable of being prepared into cross-linked compacted solids or lattice-like gels. Thus, a detailed study of the molecular structure of collagen & genes associated with each type of collagen is essential for the further research and treatment of collagen-associated disorders.
